# Savings from the introduction of BPaL and BPaLM regimens at the country level

**DOI:** 10.5588/ijtldopen.24.0213

**Published:** 2024-07-01

**Authors:** C. Auer, A. Gupta, C. Malbacius, A. Ghafoor, Y. Kock, O. Medvedieva, P. Hanlon, P. Steinmann, S. Juneja

**Affiliations:** ^1^ Swiss Tropical and Public Health Institute, Allschwil, Switzerland;; ^2^ University of Basel, Basel, Switzerland;; ^3^ TB Alliance, New York, NY, USA;; ^4^ Disease Prevention and Control Bureau, Department of Health, Manila, The Philippines;; ^5^ National Tuberculosis Program, Islamabad, Pakistan;; ^6^ National Department of Health, Pretoria, South Africa;; ^7^ Public Health Center, Ministry of Health, Kyiv, Ukraine

**Keywords:** drug-resistant TB treatment, six-month regimens, cost savings, bedaquiline, pretomanid, economic evaluation, cost analysis

## Abstract

**BACKGROUND:**

In 2022, the WHO recommended the 6-month regimens BPaL (bedaquiline + pretomanid + linezolid) and BPaLM (BPaL + moxifloxacin) as treatment options for most forms of drug-resistant TB. SLASH-TB estimates the cost-saving and cost-effectiveness for the healthcare system and patients when a country switches from current standard-of-care treatment regimens to BPaL/BPaLM.

**METHODOLOGY:**

Country data from national TB programmes (NTP) are used to calculate the costs for all regimens and treatment outcomes. Where BPaL/BPaLM is not currently used, clinical trial outcomes data are used to estimate cost-effectiveness. DALYs are calculated using the Global Burden of Disease (GBD) database.

**RESULTS:**

We present the results of four countries that have used the tool and shared their data. When shorter and longer regimens are replaced with BPaL/BPaLM, the savings per patient treated in Pakistan, the Philippines, South Africa, and Ukraine are $746, $478, $757, and $2,636, respectively. An increased number of patients would be successfully treated with BPaL/BPaLM regimens, with 411, 1,025, 1,371 and 829 lives saved and 20,179, 27,443, 33,384 and 21,924 DALYs averted annually in the four countries, respectively.

**CONCLUSION:**

Through BPaL/BPaLM regimens, drug-resistant TB treatment has become more effective, shorter, less burdensome for patients, cheaper for both health systems and patients, and saves more lives.

## RÉSUMÉ

**CONTEXTE:**

En 2022, l'OMS a préconisé l'utilisation des schémas thérapeutiques (bedaquiline + pretomanid + linezolid) et BPaLM (BPaL + moxifloxacin), d'une durée de 6 mois, comme alternatives pour traiter la plupart des formes de TB résistante aux médicaments. SLASH-TB a réalisé une estimation des économies et de la rentabilité pour le système de santé et les patients lorsqu'un pays décide de passer des schémas thérapeutiques standards actuels au BPaL/BPaLM.

**MÉTHODOLOGIE:**

Les programmes nationaux de lutte contre la TB (NTP) utilisent les données nationales pour évaluer les coûts des différents schémas thérapeutiques et des résultats des traitements. Si le BPaL/BPaLM n'est pas utilisé actuellement, les données des essais cliniques sont utilisées pour estimer le rapport coût-efficacité. Les années de vie ajustées sur l'incapacité (DALYs, pour l’anglais « *disability-adjusted life-years* ») sont calculées à l'aide de la base de données Global Burden of Disease (GBD).

**RÉSULTATS:**

Nous présentons les résultats de quatre pays qui ont utilisé l'outil et partagé leurs données. Lorsque les schémas plus courts et plus longs sont remplacés par BPaL/BPaLM, les économies par patient traité au Pakistan, aux Philippines, en Afrique du Sud et en Ukraine sont respectivement de 746, 478, 757 et 2 636 dollars. L'utilisation des schémas BPaL/BPaLM permettrait de traiter un plus grand nombre de patients avec succès, ce qui sauverait respectivement 411, 1 025, 1 371 et 829 vies et éviterait 20 179, 27 443, 33 384 et 21 924 DALYs par an dans les quatre pays.

**CONCLUSION:**

Les schémas BPaL/BPaLM ont révolutionné le traitement de la tuberculose pharmacorésistante en le rendant plus efficace, plus rapide, moins contraignant pour les patients, plus économique pour les systèmes de santé et les patients, et en sauvant un plus grand nombre de vies.

Globally, there were an estimated 410,000 incident cases of drug-resistant TB (DR-TB) in 2022. An estimated 57% of patients were not enrolled in adequate treatment for DR-TB.^1^ Apart from case finding, the cost of treatment of DR-TB patients is a key healthcare challenge for most low- and middle-income countries. Cost-saving and cost-effective DR-TB treatment options are critically needed to reduce the economic pressure on the currently strained healthcare system.

Until recently, the WHO recommended treatments for DR-TB were a 9–11-month standard short oral regimen (SSOR) and >18-month standard long oral regimen (SLOR). In 2022, the WHO updated its guidelines to recommend 6-month all-oral BPaL (bedaquiline + pretomanid + linezolid) and BPaLM (BPaL + moxifloxacin) regimens (BPaL/BPaLM) for most forms of DR-TB.^2^

Wide use of BPaL/BPaLM has the potential to substantially improve the DR-TB treatment landscape by reducing treatment costs, alleviating the burden on health systems, and saving patient-incurred costs. The Swiss Tropical and Public Health Institute and TB Alliance have developed a tool called SLASH-TB (Savings from Leveraging & Adopting Shorter & Highly Effective TB Treatments^3^) that calculates the value added from transitioning the current standard of care to the BPaL/BPaLM regimens at the country level. The tool calculates the costs and cost-effectiveness when a country continues with the standard regimens compared with when the country’s DR-TB patients are treated with BPaL/BPaLM. The tool was applied to data from four countries: Pakistan, the Philippines, South Africa, and Ukraine. This paper describes the tool and presents results from these countries.

## METHODOLOGY

### SLASH-TB and its structure

SLASH-TB compares current shorter or longer regimens with BPaLM for MDR-TB (multidrug-resistant TB) and BPaL for pre-extensively drug-resistant TB (pre-XDR-TB; MDR-TB with additional resistance to fluoroquinolones). The tool also allows for the comparison of individualised treatment regimens. The tool output shows what a country could expect regarding cost savings, cost-effectiveness, and budget impact over 5–10 years. As this was a retrospective study using data obtained from records and estimates, and no patients were interviewed, no institutional approval was required.

SLASH-TB is MS Excel-based (Microsoft, Redmond, WA, USA) for ease of application by countries where data can be provided by the National Tuberculosis Programme and/or other stakeholders. It consists of 1) guidance on how to fill the data sheets, 2) two data sheets in which the user is required to fill in country data, 3) calculation sheets, which include TB-related data extracted from the GBD databank^4^ and 4) the output dashboard.

Of the two worksheets that need to be filled, the first one, called ‘TB data’, requires data on DR-TB treatment and outcomes, such as regimens used in the previous year latest available treatment outcomes of DR-TB cohorts, and the projected number of patients in the country over the next 10 years to be treated with different regimens (BPaL, BPaLM, SSOR, SLOR). Patient-related data required for each regimen are as follows: the average number of patient visits to health facilities during treatment, the proportion of patients hospitalised and average duration of hospitalisation, daily hospitalisation costs for the health system and the patient, and other costs to calculate direct patient costs, namely minimal daily wage and average local travel costs to hospital/clinic.

The second worksheet captures the following costs for each regimen used in the country: 1) TB medicines, 2) ancillary medicines, and 3) tests for baseline and treatment monitoring (such as sputum examinations, blood tests, etc); 4) hospitalisation costs; and 5) patient costs. For the first four categories, an estimate of the proportion of patients who need them (e.g. 100% need bedaquiline under BPaL regimen, while a much lower percentage of patients may be using delamanid under the longer regimen) and the number of units needed for the treatment must be estimated. Patient costs include an average estimate for travel costs to the clinic/hospital, lost productive time (assuming minimum wages), daily hospitalisation costs borne by the patient, and other costs where available, such as childcare or caregiver costs.

When desired by a country and if data are available, other costs can be added, such as social support (cash or food baskets), capital costs, and others. SLASH-TB has the flexibility to include additional costs for both patients and service providers where such data are available.

### The treatment outcomes of SLASH-TB

For the cost-effectiveness analysis, treatment outcomes are crucial; hence, the latest available data are requested by regimen from each country. It is noted that if the country does not yet have treatment outcome data for BPaL, the treatment outcomes presented in the relevant trial (ZeNix for BPaL^5^ and TB-PRACTECAL for BPaLM^6^) are used. Data presented at The Union Conference in 2023^7^ show that countries implementing BPaL under operational research achieved similar outcomes as those in clinical trials. Treatment success is defined as the sum of patients cured and treatment completed.^8^ Patients experiencing unfavourable outcomes during post-treatment follow-up in clinical trials were not included in the assessment.

### Outputs of SLASH-TB

The outputs are presented in a table format for ease of use by countries. Table 1 presents the total costs for service providers and patients under each regimen and the average per-patient cost for each regimen. The table also presents the annual and per-patient savings for patients and providers resulting from the switch from current regimens to BPaL/BPaLM.

**Table 1.** Cost comparison for SSOR, SLOR, BPaL and BPaLM.

Regimen outcome					Savings when using BPaL/M instead of SSOR and SLOR	
	SSOR (USD)	SLOR (USD)	BPaL (USD)	BPaLM (USD)	Annual	Per patient
Pakistan						
Estimated number of patients on treatment in 2022, *n*	2,839	982	1,201	2,620		
Total cost	3,598,662	3,142,085	1,195,782	2,694,750	2,850,215	746
Per patient cost	1,268	3,200	996	1,029	42%	
Cost per successful treatment with SSOR/SLOR vs BPaL/BPaLM	2,271		1,112			
Philippines						
Estimated number of patients on treatment in 2022, *n*	5,154	2,501	161	7,494		
Total cost	9,115,023	6,507,564	246,336	11,714,494	3,661,758	478
Per patient cost	1,769	2,602	1,530	1,563	23%	
Cost per successful treatment with SSOR/SLOR vs BPaL/BPaLM	2,698		1,685			
South Africa						
Estimated number of patients on treatment in 2022, *n*	4,965	2,820	730	6,665		
Total cost	6,237,855	9,449,067	950,968	8,839,013	5,896,940	757
Per patient cost	1,256	3,351	1,303	1,328	38%	
Cost per successful treatment with SSOR/SLOR vs BPaL/BPaLM	3,268		1,437			
Ukraine						
Estimated number of patients on treatment in 2022, *n*	2,581	2,709	1,642	3,648		
Total cost	8,760,303	18,747,194	4,178,530	9,386,269	13,942,698	2,636
Per patient cost	3,394	6,920	2,545	2,573	51%	
Cost per successful treatment with SSOR/SLOR vs BPaL/BPaLM	7,969		2,739			

SSOR = standard short oral regimen; SLOR = standard long oral regimen; BPaL = bedaquiline + pretomanid + linezolid; BPaLM =BPaL + moxifloxacin.

Table 2 presents the incremental benefit for three periods: the year before the analysis and the 5- and 10-year horizons using BPaL/BPaLM. The following were calculated: successful treatment outcomes; lives saved; treatment failures averted; patients lost to follow-up averted; months of treatment saved; disability-adjusted life-years (DALYs) averted; years of life lost (YLL) due to premature mortality averted; and years lived with a disability (YLDs) averted.

**Table 2.** Incremental effectiveness of BPaL/BPaLM versus SSOR and SLOR.

Incremental effectiveness	Units	Pakistan		Philippines		South Africa		Ukraine	
		Annual (2022)	5-year (2023–2027)	Annual (2022)	5-year (2023–2027)	Annual (2022)	5-year (2023–2027)	Annual (2022)	5-year (2023–2027)
Successful treatments	Patients	530	3,187	1,307	9,187	2,015	11,330	1,501	6,546
Lives saved	Patients	411	2,454	1,025	7,007	1,371	7,010	829	3,617
Treatment failures averted	Patients	107	685	227	1,762	227	1,189	234	1,035
Patient lost to follow-up averted	Patients	320	2,176	609	4,717	1,379	7,153	708	3,082
Months of treatment saved	Months	21,447	118,650	47,771	314,184	53,471	289,143	41,838	183,948
DALYs averted	Years	20,179	133,565	27,443	214,585	33,384	164,038	21,942	93,609
YLLs averted (years of life lost due to premature mortality)	Years	19,250	127,411	25,829	201,965	31,258	153,993	20,874	89,052
YLDs averted (years lived with disability)	Years	930	6,153	1,614	12,620	2,126	10,045	1,068	4,558

BPaL = bedaquiline + pretomanid + linezolid; BPaLM =BPaL + moxifloxacin; SSOR = standard short oral regimen; SLOR = standard long oral regimen; DALY = disability-adjusted life-years; YLL = years of life lost.

A scenario analysis was conducted to explore the impact of different treatment outcomes on incremental effectiveness (Table 3). The Philippines is already using BPaL, and SLASH-TB uses the actual treatment outcomes for BPaL and TB-PRACTECAL results for BPaLM^5^ to calculate the effectiveness - captured as Scenario A. For Scenario B, we used the Zenix trial results for BPaL^4^ and interim published results for BPaLM in TB-PRACTECAL,^9^ both showing a lower treatment success rate.

**Table 3.** Scenario analysis: Incremental effectiveness for 2022, The Philippines.

				Incremental effectiveness			
	BPaL %	BPaLM %		Successful treatments *n*	Lives saved *n*	Failures averted *n*	Loss to follow-up averted *n*
Scenario A (actual data)							
Treatment success rate	98.6	92.6	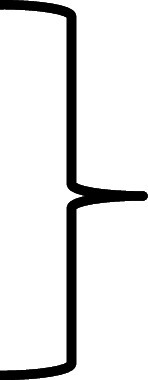	1,307			
Died	1.4	0.0			1,025		
Failure	0.0	0.0				227	
Lost to follow-up	0.0	0.0					609
Scenario B (trial data)							
Treatment success rate	89.3	88.7	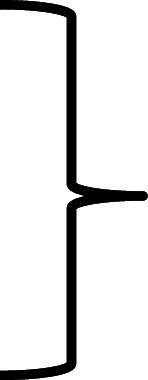	1,001			
Died	0.0	0.0			1,028		
Failure	0.6	0.0				226	
Lost to follow-up	0.6	3.2					367

BPaL = bedaquiline + pretomanid + linezolid; BPaLM =BPaL + moxifloxacin.

Table 4 presents a budget impact analysis showing cost savings for service providers and patients resulting from switching to the new regimens over 5 and 10 years.

**Table 4.** Budget impact analysis: savings when switching from SSOR and SLOR to BPaL/BPaLM.

	Pakistan		Philippines		South Africa		Ukraine	
	5-year span	10-year span	5-year span	10-year span	5-year span	10-year span	5-year span	10-year span
Number of people treated	32,812	81,481	74,946	154,216	48,069	97,119	30,551	71,512
Total savings	14,002,655	37,678,985	23,153,108	53,301,425	34,430,535	69,563,734	61,606,894	152,921,201
Provider cost saving	13,457,879	36,211,235	19,408,472	44,797,456	30,152,151	60,919,652	46,114,424	114,373,593
Patient cost saving	544,776	1,467,751	3,744,636	8,503,969	4,278,384	8,644,082	15,492,470	38,547,608
Savings per patient	427	462	309	346	716	716	2,017	2,138
Total savings, %	44	48	15	17	36	40	60	65%

SSOR = standard short oral regimen; SLOR = standard long oral regimen; BPaL = bedaquiline + pretomanid + linezolid; BPaLM = BPaL + moxifloxacin.

## RESULTS

We present the results of the four countries that have provided their data in 2023 to use SLASH-TB fully.

### Service provider and patient costs

Table 1 presents the computed costs for each country for SSOR and SLOR, as per the reported use of these regimens in 2022, stratified by total costs and costs per patient. The costs for the countries per patient ranged from $1,256 to $3,394 for SSOR and $2,602 to $6,920 for SLOR. It should be noted that the costs were the highest in Ukraine, where all patients are hospitalised for a certain period according to national treatment guidelines. If these patients were to use BPaL/BPaLM instead of SSOR or SLOR, the cost would be between $996 and $2,573 per patient, indicating savings in each country. These costs and savings are disaggregated by service provider costs and patient costs and detailed in Supplementary Data 1.

### Incremental effectiveness

In the four analysed countries, the treatment success rates for SSOR ranged between 67–81%; for SLOR, it was between 53% and 73%. In comparison, BPaL/BPaLM have ∼90% success in clinical trials and operational research in countries. Supplementary Data 2 provides detailed treatment outcome data from the four countries.

Table 2 shows that using BPaL/BPaLM, instead of SSOR and SLOR not only results in considerable cost savings but also leads to an increased number of patients annually with successful treatment outcomes (530; 1,307; 2,015; 1,501), lives saved (411; 1,025; 1,371; 829), failures averted (107; 227; 227; 234), and loss to follow-up averted (320; 609; 1,379; 708) for the four countries. Aggregate estimates for the four countries show that 3,636 lives would have been saved if these countries had been using BPaL/BPaLM in 2022, and 20,089 lives would have been saved from 2023 to 2027. The potential number of disability-adjusted life-years (DALYs) that could have been averted was considerable (20,179; 27,443; 33,384; 21,942) for the year 2022 and even more so for the next 5-year period (133,565; 214,585; 164,038; 93,609).

### Scenario analysis: Incremental effectiveness under different treatment outcome scenarios

Scenario A is the actual treatment outcomes in the Philippines for BPaL (Supplementary Data 2) and those of TB-PRACTECAL^5^ for BPaLM. Scenario B uses less favourable treatment outcomes and shows that with a lower success rate, as reported in ZeNix^4^ and interim TB-PRACTECAL trial results,^8^ the number of additional successful treatments using BPaL/BPaLM in the Philippines remains high at 1,001 compared with 1,307. Although the lives saved and failures averted remain similar due to the similarity in outcomes from various studies, the number of patients lost to follow-up increases but remains 367 fewer than when using current regimens (Table 3).

### Budget impact analysis

Over five years, depending on each country’s DR-TB burden and the speed of switch from current to new regimens, the savings are $14 million (44%), $23.2 million (15%), $34.4 million (36%), and $61.6 million (60%), respectively (Table 4).

## DISCUSSION

One of the main challenges with the tool is the collection of data that countries may not routinely gather. However, with the ongoing efforts to use WHO guidelines directly and digitise data, including the guide on ‘Electronic recording and reporting for tuberculosis care and control’,^10^ such information is becoming more readily available.

While SLASH-TB has the flexibility to include additional costs for both patients and service providers where such data are available, the flexibility also extends to unavailable data. For example, among the analysed countries, South Africa and Ukraine failed to procure data on ancillary medicines. In these cases, we removed the cost of ancillary medicines from the analysis for current regimens and BPaL/BPaLM.

SLASH-TB uses GBD country data based on TB disease and is not disaggregated for DR-TB. This likely leads to an underestimation of the burden of DR-TB disease and, hence, an underestimation of the number of lives saved through the new regimens.

The outcome ‘years lived with disability’ was adjusted by the number of patient treatment months saved when using the shorter BPaL/BPaLM regimens. In reality, the issue of disability is far more complex—TB, especially DR-TB, often results in post-TB sequelae and disabilities,^11,12^ and this is not captured adequately in SLASH-TB.

Regarding the cost borne by the patients, SLASH-TB considers the average costs of transportation, hospitalisation expenses and days of lost income due to trips to a clinic in the frame of DR-TB treatment. For lost income, the country’s minimum wage was used, likely resulting in an underestimation of lost income. Out-of-pocket expenditures are also likely underestimated for a certain proportion of patients. On the other hand, it was assumed that all patients were wage earners.

SLASH-TB also does not consider the indirect benefits of reduced duration of treatment and a lower pill burden. Long-term treatment and a high pill burden can have a strong negative impact on the patient’s quality of life. Also not considered are the potentially substantial positive effects of lower failure and relapse rates (such as the positive effects of less transmission, lower re-treatment rates, and lower re-hospitalisation rates) through the use of BPaL/BPaLM.

The evidence to support better outcomes for DR-TB when using BPaL/BPaLM regimens is impressive but not extensive. More studies on the real-world treatment outcomes of BPaL and BPaLM are currently being conducted, and additional data are expected to be published soon. Data informing SLASH-TB outcomes can be adjusted if warranted, and countries will be able to use the programmatic outcomes data when they start implementing BPaL/BPaLM.

Clinical trials and emerging data from programmatic implementation^6,13^ prove that the shorter BPaL/BPaLM regimens are more effective and more easily implementable for DR-TB. SLASH-TB calculates that implementing these treatments would lead to significant cost savings with a long-term impact on the governmental health budget, in addition to better treatment outcomes resulting in more people successfully completing treatment, fewer cases of treatment failure, and more lives saved due to the positive impact on patient cohorts. Other studies in multiple countries have also demonstrated cost savings from implementing these regimens. ^14–16^ Operational research in the Philippines shows a higher treatment success than the clinical trials on which SLASH-TB is based. However, the scenario analysis indicates that even when the worst outcomes from all trials are considered, the use of BPaL/BPaLM would still result in substantially more lives saved and fewer failures or losses to follow-ups than when continuing the SSOR and SLOR; thus, these interventions would remain highly cost-effective. Therefore, there is a strong economic and treatment outcome-based case for the rapid implementation of BPaL/BPaLM regimens. The expected monetary savings could be used to improve diagnostic access and treatment support. This will contribute to closing the enormous gap that exists between the incidence cases of DR-TB and those enrolled in adequate treatment: only approximately 43% of the estimated number of people who developed DR-TB in 2022 were enrolled in treatment.^1^

## CONCLUSION

By implementing the BPaL/BPaLM regimens, DR-TB treatment has become more effective, shorter, less burdensome for patients, and cheaper for both health systems and patients. SLASH-TB expresses this in numbers; therefore, it is helpful for country planning, advocacy, and budgeting. Countries will benefit from using SLASH-TB for planning purposes and advocacy by presenting concrete figures that show the benefit of the newly recommended BPaL/BPaLM regimens. The results from SLASH-TB support the case for rapid implementation of BPaL/BPaLM made in published advocacy documents, such as the Call to Action of WHO and partners^17^ and the Global Fund’s advocacy guide on six-month treatments for DR-TB.^18^

The main challenge now is to scale up the use of BPaL/BPaLM regimens rapidly. There is an epidemiological, economic, and moral imperative to adequately support countries in these scaling-up efforts. SLASH-TB provides countries with an accessible tool to rapidly conduct cost-effectiveness and budget impact analyses to support policy decisions, at no to minimal cost. TB Alliance intends to disseminate information on the tool widely so that countries worldwide can benefit from it.

## Supplementary Material


