# Supplementary material for: Savings from the introduction of BPaL and BPaLM regimens at the country level
Source: IJTLD Open. 2024 Jul 1;1(7):314–9. doi: 10.5588/ijtldopen.24.0213 (PMC11257096; doi:10.5588/ijtldopen.24.0213)
Supplement: Supplementary file 1 [file ijtldopen0213_supplementarydata1.pdf]

http://dx.doi.org/10.5588/ijtldopen.24.0213

SUPPLEMENTARY DATA

Savings from the introduction of BPaL and BPaLM regimens at the country level

Supplementary Table S1. Detailed cost data of all regimens from the four study countries

Regimen	SSOR	SLOR	BPaL	BPaLM	Savings when using BPaL/BPaLM instead of SSOR and SLOR	
**PAKISTAN**						
**Estimated number of patients on treatment**	2’839	982	1’201	2’620	Annual Saving	Saving per patient
**Service Provider Costs**	3’403’083	3’030’986	1’130’700	2’552’772	2’750’598	720
TB Medicines	*1’848’327*	*2’003’167*	*694’610*	*1’601’134*	*1’555’750*	*407*
Ancillary Medicines	*4’161*	*6’869*	*356*	*2’254*	*8’420*	*2*
Baseline testing & Monitoring	*424’960*	*244’207*	*116’676*	*253’354*	*299’137*	*78*
Hospitalisation	*11’356*	*5’892*	*4’804*	*10’480*	*1’964*	*0.5*
Social support	*1’114’279*	*770’850*	*314’254*	*685’549*	*885’327*	*232*
**Patient Costs**	195’579	111’099	65’082	141’978	99’617	26
**Total Costs**	3’598’662	3’142’085	1’195’782	2’694’750	2’850’215	745.9
Per patient cost	*1’268*	*3’200*	*996*	*1’029*	*42%*	
**PHILIPPINES**						
**Estimated number of patients on treatment**	5’154	2’501	161	7’494	Annual Saving	Saving per patient
**Service Provider Costs**	8’459’087	5’953’445	232’630	11’076’559	3’103’341	405
**TB Medicines**	2’780’094	2’257’935	91’503	4’470’544	475’982	62
**Ancillary Medicines**	171’544	182’884	5’218	279’889	69’322	9
**Baseline testing & Monitoring**	5’481’678	3’497’620	135’266	6’296’151	2’547’882	333
**Hospitalisation**	25’770	15’006	644	29’976	10’156	1
**Patient Costs**	655’937	554’119	13’705	637’934	558’416	73
**Total Costs**	9’115’023	6’507’564	246’336	11’714’494	3’661’758	478
Per patient cost	*1’769*	*2’602*	*1’530*	*1’563*	*23%*	
**SOUTH AFRICA**						
**Estimated number of patients on treatment**	4’965	2’820	730	6’655	Annual Saving	Saving per patient
**Service Provider Costs**	5’257’318	8’504’084	839’885	7’826’331	5’095’186	654
**TB Medicines**	3’171’543	6’379’374	555’355	5’232’427	3’763’134	483
**Ancillary Medicines**	-	-	-	-	-	-
**Baseline testing & Monitoring**	1’965’126	1’953’395	266’791	2’432’188	1’219’543	157
**Hospitalisation**	120’650	171’315	17’739	161’717	112’509	14
**Patient Costs**	980’536	944’983	111’083	1’012’682	801’754	103
**Total Costs**	6’237’855	9’449’067	950’968	8’839’013	5’896’940	757
Per patient cost	*1’256*	*3’351*	*1’303*	*1’328*	*38%*	
**UKRAINE**						
**Estimated number of patients on treatment**	2’581	2’709	1’642	3’648	Annual Saving	Saving per patient
**Service Provider Costs**	6’525’372	14’269’540	3’187’426	7’184’354	10’423’132	1’970
**TB Medicines**	1’376’836	4’017’807	926’684	2’161’707	2’306’252	436
**Ancillary Medicines**	-	-	-	-	-	-
**Baseline testing & Monitoring**	1’896’409	3’424’913	881’434	1’958’264	2’481’624	469
**Hospitalisation**	3’252’127	6’826’820	1’379’308	3’064’383	5’635’255	1’065
**Patient Costs**	2’234’931	4’477’655	991’103	2’201’915	3’519’567	665
**Total Costs**	8’760’303	18’747’194	4’178’530	9’386’269	13’942’698	2’636
Per patient cost	*3’394*	*6’920*	*2’545*	*2’573*	*51%*	

Supplementary Table S2. Treatment outcomes of the short and the long regimens in the four included countries

Country	*Treatment outcome*	*Short regimen(s)*	*Long regimen(s)*	*BPaL* [a]
**Pakistan**	*Cohort size*	*n = 468*	*n = 1715*	
	Successful treatment	79.5% (372)	72.5% (1243)	
	Failure	3.0% (14)	3.0% (52)	
	Died	8.1% (38)	18.4% (315)	
	Lost to follow-up	9.4% (44)	6.1% (105)	
**Philippines**	*Cohort size*	*n = 4247*	*n = 1124*	*n = 73*
	Successful treatment	80.9% (3437)	64.8% (728)	98.6% (72)
	Failure	2.9% (123)	3.1% (35)	0
	Died	8.5% (359)	23.7% (266)	1.4% (1)
	Lost to follow-up	7.7% (328)	8.5% (95)	0
**South Africa**	*Cohort size*	*n = 4096*	*n = 2040*	
	Successful treatment	66.6% (2728)	52.9% (1080)	
	Failure	2.0% (80)	4.8.% (97)	
	Died	16.5% (677)	19.5% (398)	
	Lost to follow-up	14.9% (611)	22.8% (465)	
**Ukraine**	*Cohort size*	*n = 1719*	*n = 2754*	*n = 98*
	Successful treatment	78.6% (1351)	52.5% (1447)	95.9% (94)
	Failure	2.1% (36)	7.3% (200)	1.0% (1)
	Died	9.0% (154)	22.1% (608)	0
	Lost to follow-up	10.4% (178)	18.1% (499)	3.1% (3)

[a] At the time of this analysis, the Philippines and Ukraine were able to share the treatment outcome results for BPaL. None of the above countries were able to share any data for BPaLM.

For countries that do not yet have treatment outcome data for BPaL, the following was used for the model (based on the ZeNix trial): Treatment success of 89.3%, failed 0.6%, died 0.0%, lost to follow-up 0.6%. For BPaLM, the following was used (based on the TB-Practecal trial): Treatment success of 92.6%, failed 0.0%, died 0.0%, lost to follow-up 0.0%.

References:
- ZeNix: https://pubmed.ncbi.nlm.nih.gov/36053506
- TB-PRACTECAL: https://www.thelancet.com/journals/lanres/article/PIIS2213-2600(23)00389-2/fulltext
